# Lumbar Morel-Lavallée lesion: a case report and review of the literature

**DOI:** 10.1186/s13256-023-03922-0

**Published:** 2023-05-03

**Authors:** Michèle Yolande Moune, Ben Ousmanou Djoubairou, Fred Mboka, Yadji Viche, Abdessamad El Ouahabi

**Affiliations:** 1 Department of Neurosurgery, Hôpital Des Spécialités, CHU de Rabat, Rabat, Morocco; 2 Yaounde Military Hospital, Yaounde, Cameroon

**Keywords:** Lumbar Morel-Lavallée lesion, Woman, Management, Case report

## Abstract

**Objectives:**

Here we describe a rare post-traumatic lesion and discuss its management.

**Background:**

Lumbar Morel-Lavallée is a rarely reported lesion. The cause is usually post-traumatic in a polytraumatic context, and care is often focused elsewhere. This leads to misdiagnosis with a risk of chronic pain and infection. In addition, there is no consensus for the management as few cases have been reported so far.

**Case report:**

A 35-year-old African woman was involved in a motor accident. Physical examination at the emergency department revealed moderate head trauma, a lumbar inflammatory mass, and a closed leg fracture. She underwent a whole-body computed tomography scan, which revealed a left frontal brain contusion and a large left paraspinal mass in favor of a lumbar Morel-Lavallée lesion. She benefited from osteosynthesis and conservative management of the cerebral and lumbar lesions. After 4 days, she complained of headaches and vomiting. Magnetic resonance imaging was requested. There was resorption of the cerebral contusion, and the lumbar mass was heterogeneous. She was discharged 10 days later without lower back pain and fully recovered from the headaches. Ultrasound of the lumbar soft tissue performed a month later showed no more collection.

**Conclusion:**

More frequent in young men, lumbar Morel-Lavallée lesion is underdiagnosed. Thus, there is no consensus on its treatment. However, conservative management followed by close monitoring is advisable in the acute phase. Other therapy includes surgery with or without the use of sclerosing agents. Early diagnosis prevents infections. Although the diagnosis is clinical, magnetic resonance imaging is the critical paraclinical examination for its assessment. Our case is interesting because it occurs in a woman following polytrauma, and to the best of our knowledge, it is an extremely rare lesion, especially in women.

## Background

Lumbar Morel-Lavallée is a rarely reported lesion. The etiology is post-traumatic due to the shear force that separates the underlying fascia from the subcutaneous tissue. The space created is filled with blood, lymph, and necrotic fat. It generally occurs in the context of polytrauma, and care is often directed elsewhere. It is, therefore, underdiagnosed with a risk of chronic pain and infection. Although rare, it is an unusual cause of hemorrhagic shock.

## Case study

**Fig. 1:**
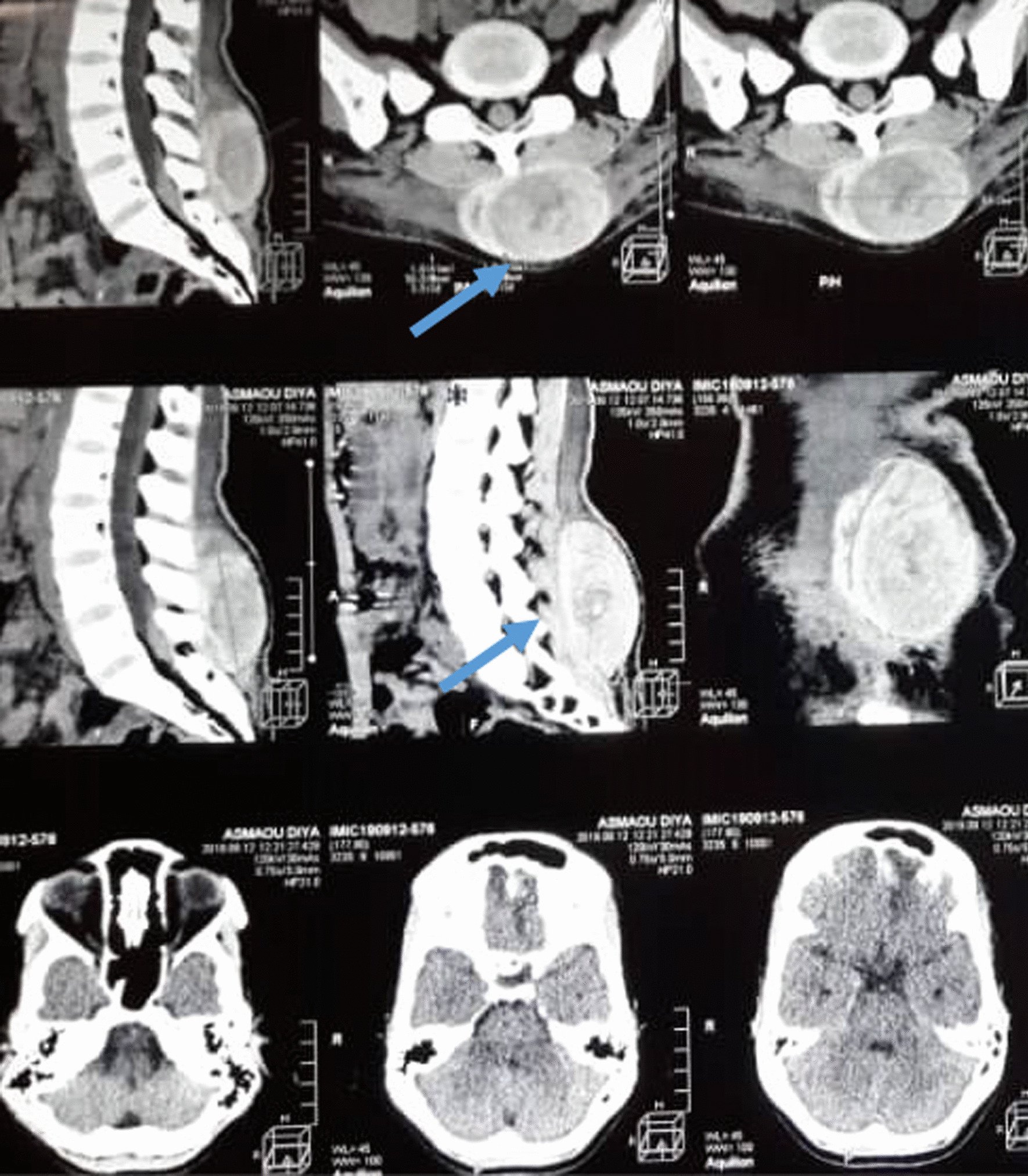
Cerebral CT: left frontal concussion lumbar. CT: left paraspinal hyperdense mass. The arrows are pointing the Morel-Lavallée Lesion

A 35-year-old African woman without medical or surgical history was involved in a motor accident. She complained of pain in her lower back and left leg. She was initially admitted to a hospital with no neurosurgery department, underwent a full body scan, and was then transferred to our center. Physical examination at the emergency department 6 hours after the accident revealed normal vital signs with respiratory rates of 16 breaths per minute, 100% oxygen saturation on ambient air, heart rate of 86 beats per minute, blood pressure of 122/78 mmHg, and temperature of 36 °C. The Glasgow Coma Scale was 14 (motor 6, verbal 5, and eyes 3), and the pupils were symmetric and reactive to light. Neurologic examination showed no deficit except on the left leg due to a closed fracture. A back examination revealed a large swelling on the lower back without a skin lesion. The rest of the physical examination was unremarkable. Whole-body computed tomography (CT) scan showed a left frontal contusion with no mass effect. However, a voluminous left hyperdense paraspinal mass indicated a possible lumbar Morel-Lavallée lesion (Fig. 1). There was no associated vertebral fracture or sign of a spinal cord lesion. She underwent osteosynthesis for the leg fracture immediately after admission and conservative management of the cerebral and lumbar lesions. The management consisted of painkillers, sodium valproate for the brain contusion, and compressive bandage with bed rest for the Morel-Lavallée lesion.

**Fig. 2:**
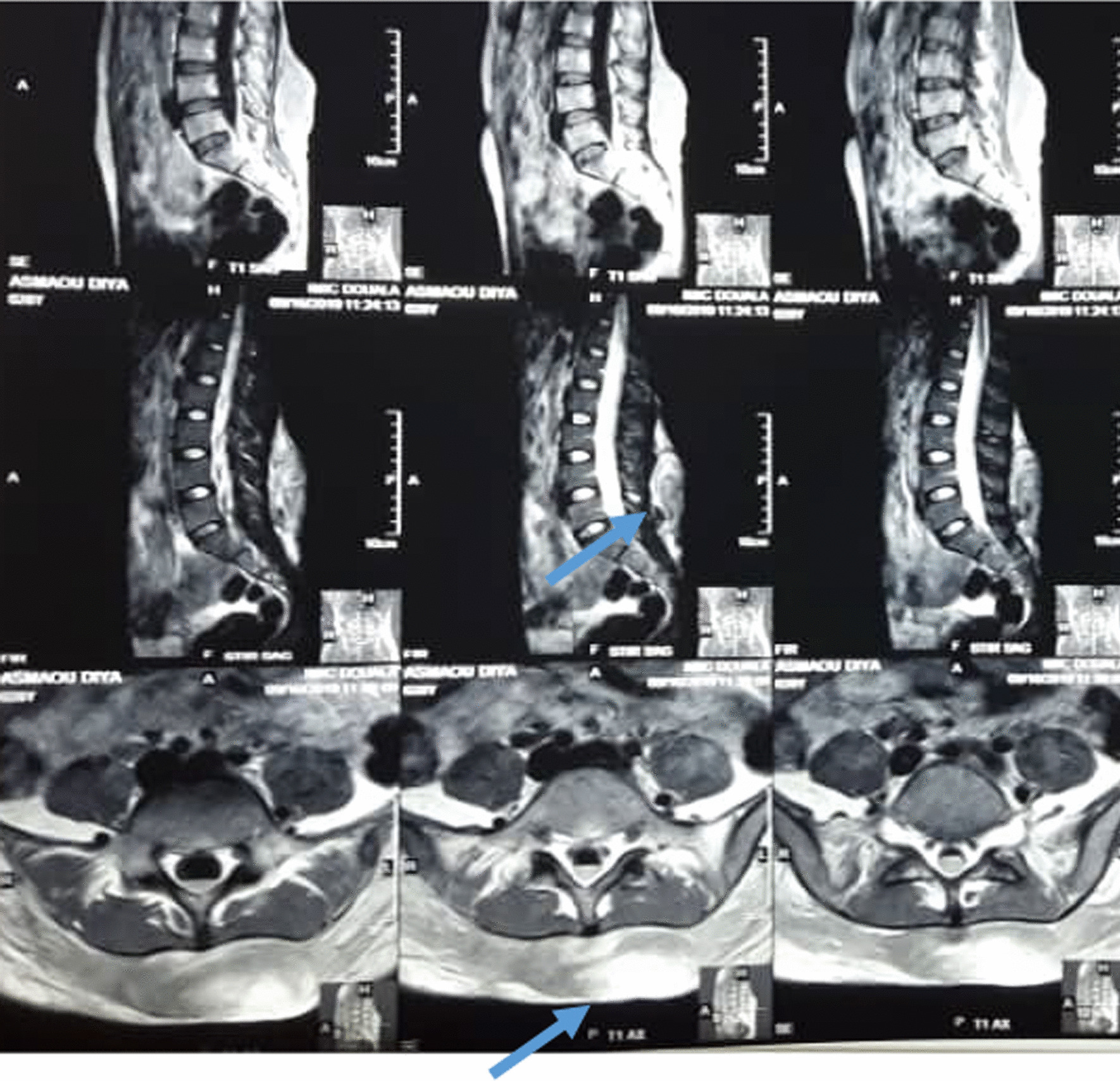
Heterogeneous hyperintense left paraspinal mass on T1 and T2. The arrows are pointing the Morel-Lavallée Lesion

After 4 days, she complained of headaches and vomiting, and magnetic resonance imaging (MRI) was conducted in the hospital. Unfortunately, our hospital has no access to CT scans; only MRI is available. MRI revealed resorption of the cerebral contusion, and the lumbar mass appeared heterogeneous in both T1 and T2 sequences (Fig. 2). No additional treatment was added, and she was discharged 10 days later without headaches or lumbar pain and with good evolution of the leg fracture. Ultrasound of the lumbar soft tissue performed a month later was normal.

## Discussion

Morel-Lavallée lesion is rarely reported, especially in lumbar localization, where the incidence is 3.4% [3]. Other localizations include hip (30.4%), thigh (20.1%), pelvis (18.6%), knee (15.7%), gluteal (6.4%), abdominal wall (1.5%), calf (1.5%), head (0.5%), and unspecified 2% [14]. There is a male predominance with a sex ratio of male to female 2:1, probably related to the high percentage of men involved in polytrauma [1].

The etiology is post-traumatic, with shear forces that separate the underlying fascia from the subcutaneous tissue. The result is an injury of the trans-aponeurotic capillaries and lymphatics, leading to the collection of a hemolymphatic mass in the space created [3, 13]. The rate at which this collection forms depends on the number of vessels and the involvement of lymphatics versus arterial beds.

Patients usually present within hours to days after the trauma, but up to one-third after months or years [3]. Pain and swelling are the main complaints, but patients can also experience loss of cutaneous sensation due to the injury of subdermal afferent nerves [5]. A physical examination can find dermal changes such as dying, cracking, discoloration, or necrosis at advanced stages. Other clinical findings include skin hypermobility and compressible and fluctuant mass [5, 14]. In rare cases, it can cause life-threatening hemorrhagic shock.

Morel-Lavallée can occur in isolation but is usually associated with an underlying fracture [3]. Ultrasound and radiographs can identify the lesion; however, the lesion must have a significant size since the radiographs [3] and the ultrasound are operator dependent and cannot be performed in the case of an open wound and dressing [5]. The aspect of this lesion varies with time due to internal blood product degeneration, making it difficult to detect. These lesions are often mistaken for soft tissue tumors such as sarcoma, fat necrosis, pseudo lipomas, or abscess, leading to management delays. CT scan is usually performed at the emergency unit for traumatic injuries but poses the problem of irradiation, and soft tissue is not readily evaluated [5, 8]. CT image findings depend on the delay from trauma, with acute lesions having a density of 15–40 Hounsfield units, slightly lower than blood density due to the mix of blood and lymphatic fluid [5, 8]. A CT scan can also identify associated fractures and evaluate other associated lesions, especially in a polytraumatic context. MRI is the examination of choice, although it is not available in all trauma centers and has some technical limitations.

In 2005, a classification of Morel-Lavallée lesion was established according to the delay of trauma, amount of blood fat and lymph, contrast enhancement, and presence of a capsule. Other than giving a detailed characterization of the lesion, this classification does not provide guidance or the outcome of each class [13].

There are currently no guidelines for managing this lesion due to its rarity. Conservative management is advisable in the acute phase but is not always achievable, especially when faced with polytrauma. It consists of compression bandaging, pain killer, and bed rest. Bed rest allows spontaneous tamponade of the bleeding due to compression from the patient’s body weight. This therapeutic option usually minimizes the localizations outside the knee and must be associated with another approach [13]. Other options are percutaneous aspiration, sclerosis, and open drainage with irrigation.

Complications are mainly infectious with tissue necrosis, and data regarding bacterial colonization are inconclusive [13]. In addition, the aesthetic aspect may be a complaint in the long term, although the voluminous lesion was only responsible for pain in our patient. However, no studies have found a relationship between volume of this lesion and outcome.

Table 1 summarizes the cases reported over the past 10 years.

**Table 1:** Summary of all reported cases of lumbar Morel-Lavallée lesion

Author	Age	Sex	Mechanism	Delay	Treatment	Outcome
Sawkar (2011) [11]	16 18	Male Male	MVA MVA	Acute 3 months	Aspiration ?	Good ?
Efrimescu (2012) [6]	14	Male	Fall	Acute	Open drainage	Good
Moran (2012) [10]	20	Male	Sport	2 weeks	Aspiration	Good
Garisson (2014) [7]	21	Male	Sport	Acute	Conservative	Good
Bommie (2014) [12]	22	Male	MVA	6 months	Debridement	Good
Andersen (2014) [2]	19	Female	Fall	2 years	?	?
Zairi (2015) [14]	50	Male	Fall	2 years	Debridement	Good
Buyukkaya (2015) [4]	18 56	Male Male	MVA Fall	3 months Acute	Conservative Drainage	? ?
Mooney (2019) [9]	48	Female	Fall	1 month	Open drainage	Good

*MVA* motor vehicle accident

## Conclusion

Lumbar Morel-Lavallée lesion is a rare lesion with a clinical diagnosis, but MRI is the gold standard for its assessment. Conservative management in the acute phase is advisable with a good outcome. Physicians should consider this diagnosis when a patient presents with high-velocity, blunt trauma injury with persistent pain.

## Data Availability

Not applicable.
